# Ritodrine Should Be Carefully Administered during Antenatal Glucocorticoid Therapy Even in Nondiabetic Pregnancies

**DOI:** 10.1155/2013/120735

**Published:** 2013-02-28

**Authors:** Masaki Ogawa, Yoshio Matsuda, Aiko Kobayashi, Etsuko Shimada, Yoshika Akizawa, Minoru Mitani, Yasuo Makino, Hideo Matsui

**Affiliations:** ^1^Perinatal Medical Center, Tokyo Women's Medical University Hospital, Kawadacho 8-1, Shinjuku, Tokyo 162-8666, Japan; ^2^Department of Obstetrics and Gynecology, Tokyo Women's Medical University, Tokyo, Japan

## Abstract

*Aim*. Antenatal glucocorticoid therapy (AGT) has been commonly used recently. However, this therapy has severe harmful effects such as maternal hyperglycemia. In Japan, ritodrine hydrochloride has been used as a tocolytic agent. In this study, we performed retrospective casecontrol study to clarify whether concomitant use of ritodrine and glucocorticoid was safe to pregnant women without diabetes mellitus. *Methods*. We reviewed the computerized records of pregnant women with pregestational diabetes (*n* = 9) and nondiabetes (*n* = 45) who gave birth at our hospital between 2002 and 2011. Cases and controls received AGT. Blood glucose after the therapy was analyzed, and additional volume of insulin was compared to that before the therapy. *Results*. From this study, 30 units of insulin were necessary when performing AGT in diabetic pregnant women. And also, an increase in blood glucose of 40 mg/dL was seen after the therapy even in nondiabetic pregnant women. Blood glucose increased significantly in the group that also received ritodrine, and it was shown that the number of pregnant women who might develop ketoacidosis might increase 11-fold. *Conclusions*. Ritodrine should be carefully administered during antenatal glucocorticoid therapy. It may be necessary to adequately monitor blood glucose, when performing the therapy, even in nondiabetic pregnant women.

## 1. Introduction

Antenatal glucocorticoid therapy (AGT) has been a matter of worldwide debate since an epoch-making report by Liggins and Howie [1]. However, it has been commonly used in Japan recently because of clear evidence of prevention of respiratory distress syndrome in preterm infants [2–4]. This therapy has severe harmful effects such as maternal hyperglycemia [5, 6]. Endocrinologists and perinatologists must pay attention to the blood glucose levels of diabetic patients undergoing AGT. In some patients with diabetes mellitus, additional insulin therapy is needed. Unfortunately, there are no guidelines on additional insulin therapy, and little is known about the suitable volume of additional insulin.

Many patients requiring AGT present as threatened preterm labor, with regular uterine contractions and/or progressive uterine cervical dilatation. Subsequently, they also need tocolytic agents. Tocolytic therapy in Japan consists of two main drugs, ritodrine hydrochloride and magnesium sulfate, unlike many Western countries. Ritodrine, the main traditional drug in Japan, is a beta-2 stimulant that causes maternal hyperglycemia. Using both ritodrine and glucocorticoid could cause maternal hyperglycemia. However, there are no guidelines for cases like this without diabetes mellitus. Star et al. reported that prophylactic use of insulin was better [7], but Schumacher et al. opposed this notion [8]. It is very difficult to extrapolate from these overseas reports why substantial tocolytic therapy in Japan is different from that in those other countries.

We planned this retrospective case-control study to clarify what volume of additional insulin is needed for preventing maternal hyperglycemia in diabetic patients undergoing AGT and to clarify how nondiabetic patients requiring tocolytic agents after the therapy should be managed.

## 2. Materials and Methods

This study was approved by our institutional ethics committee. We reviewed the computerized records of pregnant women with pregestational diabetes mellitus (*n* = 9) and uncomplicated pregnant women (*n* = 45) with singleton gestations who gave birth at Tokyo Women's Medical University Hospital between January 2002 and March 2011. Cases and controls received AGT after giving full informed consent based on the guidelines of our institutional review board. Type 1, type 2, and gestational diabetes mellitus in the previous pregnancy were treated as pregestational diabetes mellitus. Cases without pregnancy prolongation less than 72 hours after AGT were excluded from this study. Fetal malformation and fetal abnormal karyotyping were also excluded.

AGT was performed according to the original protocol by Liggins and Howie [1]. In brief, two doses of 12 milligrams of betamethasone were injected intramuscularly 24 hours apart. Blood was sampled at four points: before injection of betamethasone (Day 0) and one day (Day 1), two days (Day 2), and three days (Day 3) after the injection. The last three points were fasting blood glucose before the morning meal. In cases of pregestational diabetes mellitus, insulin was administered subcutaneously in order to maintain blood glucose at less than 100 mg/dL before meals and/or less than 120 mg/dL two hours after meals. Ritodrine hydrochloride (100 *μ*g/hour IV) and/or magnesium sulfate (1 gram/hour IV) were used as tocolytic agents according to the previously reported method [9].

Statistical analyses were performed with a computer program: Statflex 6.0 (Artech Co., Ltd., Osaka, Japan. URL: http://www.statflex.net/). Values were given as means ± SD. Mann-Whitney *U* test, two-factor analysis of variance, and Friedman test were performed after Bartlett test. Dunn's test and Scheffe's test were performed as multiple comparisons. Spearman's rank test was performed to clarify the correlation coefficient. *χ*-square test was performed to clarify the odds ratio with 95% confidence intervals. A *P* value less than 0.05 was considered to be significant.

## 3. Results

**Figure 1:**
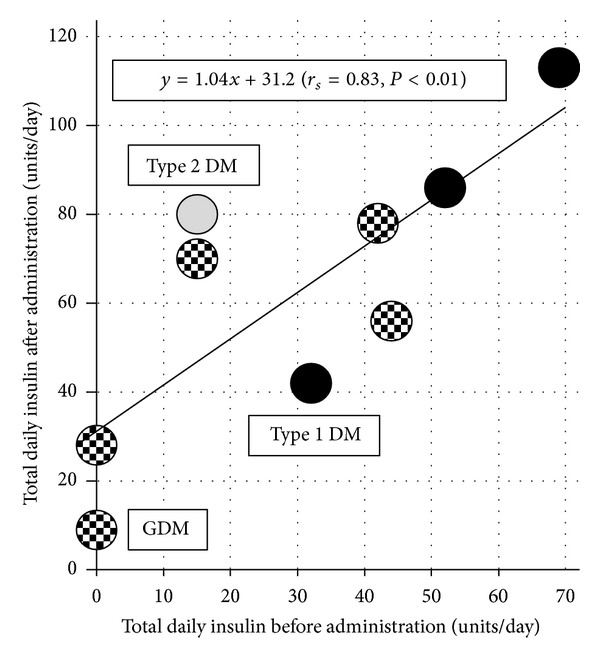
Distribution diagram of daily insulin volume before/after antenatal glucocorticoid administration. Closed circle: type 1 diabetes mellitus, open circle: type 2 diabetes mellitus, and dotted circle: gestational diabetes mellitus. Correlation coefficient by Spearman's rank test was 0.83 (*P* < 0.01). DM: diabetes mellitus, GDM: gestational diabetes mellitus.

**Figure 2:**
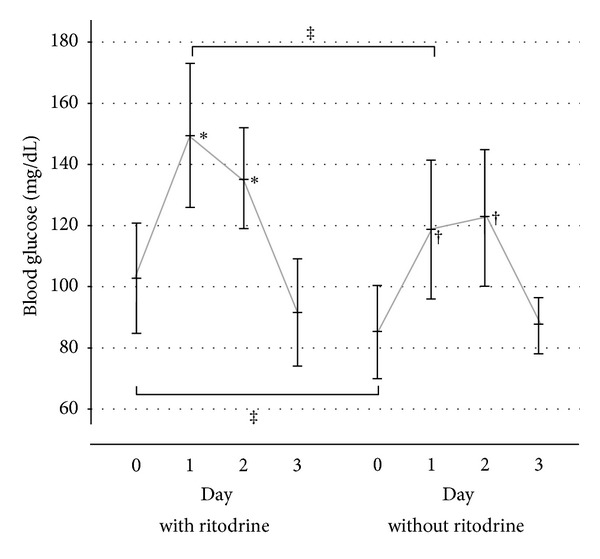
The time course of blood glucose in controls that received or did not receive ritodrine tocolysis. Asterisk (∗, †) indicates statistical significance (*P* < 0.05) versus Day 0. Asterisk (‡) indicates statistical significance (*P* < 0.05).

Cases were defined as pregestational diabetes mellitus and gestational diabetes mellitus in the previous pregnancy. The mean age was 32.8 ± 2.2 years old. Three cases of type 1 diabetes mellitus, one case of type 2 diabetes mellitus, and five cases of gestational diabetes mellitus were included in this study. The gestational weeks of AGT were 29.0 ± 3.0. Delivery weeks were 29.7 ± 2.9. Total daily insulin volume before or after AGT was 29.9 ± 23.0 or 62.4 ± 30.3, respectively. Figure 1 shows the distribution diagram of daily insulin volume before/after administration. Controls were defined as having no evidence of diabetes mellitus. The mean age of controls was 32.2 ± 4.6 years old. The gestational weeks of AGT were 29.0 ± 3.6. Delivery weeks were 31.2 ± 3.9. Thirty-seven controls received ritodrine tocolysis, 20 received magnesium tocolysis, and 17 were treated with both. As the time course of blood glucose in controls, Friedman test and subsequent Dunn's test revealed significant increases on Day 1 (143.9 ± 26.0, *P* < 0.01) and Day 2 (132.9 ± 17.9, *P* < 0.01) but not Day 3 (90.9 ± 16.3) relative to Day 0 (99.7 ± 18.6). Figure 2 shows the time course of blood glucose in controls that received or did not receive ritodrine tocolysis. Two-factor analysis of variance and subsequent Scheffe's test revealed significant increases on Day 1 (149.4 ± 23.6, 118.4 ± 22.4, *P* < 0.05) and Day 2 (135.2 ± 16.4, 122.5 ± 21.7, *P* < 0.05) but not Day 3 (91.6 ± 17.5, 87.8 ± 9.0) relative to Day 0 (102.8 ± 18.0, 85.4 ± 14.6, resp.) in controls that received or did not receive ritodrine. Blood glucose on Day 0 and Day 1 in the controls that received ritodrine was significantly higher than that in those that did not receive ritodrine (*P* < 0.05). In Table 1, the ratio of patients with blood glucose above 120 after AGT increased significantly on Day 1 in the group that used ritodrine as compared with the group that did not use ritodrine (odds ratio: 11.3, 95% confidence interval: 2.28–56.4).

## 4. Discussion

AGT is used in Japan because it lowers the incidence of respiratory distress syndrome and periventricular leukomalacia in premature infants [10]. Its use has increased recently due to the fact that it is now covered by insurance. On the other hand, maternal hyperglycemia is sometimes found as a serious side effect of AGT [11], and it causes secondary ketoacidosis [6]. Maternal acidosis is a critical problem for the child [12]. Consequently, endocrinologists and obstetricians are very sensitive to glycemic control after AGT in pregnant women with diabetes mellitus. However, there are no guidelines that give an idea of the amount of insulin needed. Moreover, how to manage nondiabetic pregnant women during AGT is unclear. Cases that require AGT are those with threatened premature labor or pregnancy induced-hypertension, and delivery must be prolonged for 48 hours, the time it takes for the effects of AGT to appear. To this end, attempts are often made to forcibly suppress labor after AGT. In Japan, there is a tendency to use ritodrine hydrochloride, a beta-2 stimulant that readily changes blood glucose, to suppress labor in such cases. However, there have been no previous reports in the literature concerning management of blood glucose while using ritodrine hydrochloride during AGT. Overseas, magnesium sulfate is used to suppress labor, not ritodrine hydrochloride. Therefore, extrapolating overseas management of blood glucose during AGT directly to Japan would carry a large risk. Investigations unique to circumstances in Japan are necessary. The present study was conceived and planned based on these circumstances.

The results of the present study showed that 30 units/day of insulin were necessary in pregnant women with diabetes mellitus receiving AGT. This includes pregnant women who did not use insulin before AGT. We could not evaluate the amount of insulin necessary by type of diabetes mellitus due to the small sample size. However, the results suggested that those with type I diabetes mellitus required more insulin, due to the fact that the slope of the regression line was steeper. In pregnant women with diabetes mellitus, it was clear that AGT easily caused hyperglycemia. In the present investigation, the additional insulin was administered by subcutaneous bolus injection, but administration by continuous subcutaneous insulin infusion (CSII) should also be investigated in the future. However, as pointed out by Bouhanick et al. [13], CSII in pregnant women with type 1 diabetes mellitus can cause decreased compliance in association with decreased subcutaneous blood flow, easily putting them at risk for ketoacidosis. Therefore, thorough investigation may be necessary before its widespread clinical application.

In nondiabetic pregnant women, as well, early morning fasting blood glucose after AGT increased at least 40 mg/dL on average. Furthermore, it was found that this hyperglycemia continued for 2 days. This hyperglycemia increased significantly in the group that used ritodrine, and the proportion of pregnant women in the ritodrine group whose early morning fasting blood glucose exceeded 120 mg/dL was 90%. The risk of developing hyperglycemia ≥120 mg/dL early in the morning while fasting on Day 1 after AGT was 11 times greater with use of ritodrine than without use of ritodrine. Fisher et al. reported that pregnant women were more prone to developing glucose intolerance during concomitant use of the beta stimulant oral terbutaline and AGT [5]. Bernstein and Catalano reported a case of diabetic ketoacidosis during concomitant use of terbutaline and AGT in a pregnant woman with normal glucose tolerance [14]. Based on the previous findings, concomitant use of AGT and a beta stimulant might increase the risk of glucose intolerance and diabetic ketoacidosis even in nondiabetic pregnant women. In particular, the fact that diabetic ketoacidosis occurred in 4/11 (36%) diabetic pregnant women with a blood glucose level <200 mg/dL shows that adequate attention should be paid when performing AGT [15].

In conclusion, this study showed that 30 units of insulin were necessary when performing AGT in pregnant women with diabetes mellitus. In addition, an increase in blood glucose of 40 mg/dL was seen after AGT even in nondiabetic pregnant women. Blood glucose increased significantly in the group that also received ritodrine, and it was shown that the number of pregnant women who might develop ketoacidosis might increase 11-fold. Therefore, it may be necessary to adequately monitor blood glucose, when performing AGT, even in nondiabetic pregnant women.

## Figures and Tables

**Table 1:** Ratios of patients with blood glucose above 120 after antenatal glucocorticoid therapy.

	Day 1			Day 2			Day 3			
	<120	120≤	P*	<120	120≤	P*	<120	120≤	P*	
Without ritodrine	4	4	0.003	3	5	0.108	8	0	0.501	8
With ritodrine	3	34		5	32		35	2		37

	7	38		8	37		43	2		45

*χ-square test.
